# The impact of the COVID-19 pandemic on obsessive compulsive disorder: a single case study

**DOI:** 10.1192/bjo.2021.350

**Published:** 2021-06-18

**Authors:** Josephine Sibanda-Mbanga, Anusha Govender

**Affiliations:** South West London & St George's Mental Health NHS Trust

## Abstract

**Objective:**

A growing body of research evidence shows that individuals with Obsessive Compulsive Disorder (OCD) have been negatively affected by the COVID-19 pandemic including deterioration of OCD symptoms plus relapse from previously well-controlled OCD. The impact of the COVID-19 pandemic is discussed in a single case study of a patient with OCD consisting of contamination concerns. In addition, the effectiveness of providing Exposure and Response Prevention (ERP) virtually is evaluated with regards to the treatment outcome in COVID-19 related OCD.

**Case report:**

This study describes a 41-year-old, single, employed female with OCD consisting of concerns of contamination and infecting others thereby causing harm. The total duration of the disorder is 32 years with the most recent presentation being of three years duration. Relapse during the pandemic resulted in OCD symptoms being solely concerned with COVID-related contamination. The questionnaires routinely completed at the time of assessment and treatment were the Obsessive Compulsive Inventory (OCI); Yale Brown Obsessive Compulsive Scale (YBOCS); Beck Depression Inventory (BDI). Clinical data were collated and analysed prior to and during the pandemic. Treatment consisted of ERP and was adapted for provision via a virtual platform. ERP involved exposure to a graded hierarchy of COVID-specific anxiety-provoking situations modified to take government guidelines into consideration.

**Discussion:**

Prior to the COVID-19 pandemic the patient's response to treatment with cognitive behavioural therapy (CBT) including ERP indicated a 79% improvement in OCD symptoms on self -rated measures. The impact of the pandemic led to a significant 65% deterioration in OCD symptoms, regarding COVID-19 contamination concerns. Intervention with ERP resulted in 73% improvement over a three-month period. Measures of depression symptoms indicated an 80% improvement pre-COVID, with a 78% deterioration at relapse. Following treatment, the patient also showed a 65% improvement in depression symptoms. Improvements have been maintained at one month follow-up.

**Conclusion:**

The case study supports literature indicating the exacerbation of OCD symptoms due to the COVID-19 pandemic for patients with contamination fears and washing compulsions. The promising results support the use of ERP as an effective treatment for COVID-related OCD symptoms. It also validates the provision of CBT interventions virtually to ensure accessibility of treatment to OCD sufferers.

